# Case Report and review of the literature: A rare cut – surgical resection of primary CNS EBV- positive T-cell lymphoma

**DOI:** 10.3389/fonc.2025.1638461

**Published:** 2025-08-27

**Authors:** Lykourgos Anastasopoulos, Eirini Charalampopoulou, Theodoros Argyrakos, Fotis Bourlogiannis, Stefanos Korfias

**Affiliations:** ^1^ 1st University Department of Neurosurgery, General Hospital of Athens Evangelismos, Athens, Greece; ^2^ Athens Microneurosurgery Laboratory, Evangelismos Hospital, Athens, Greece; ^3^ Department of Pathology, General Hospital of Athens Evangelismos, Athens, Greece

**Keywords:** primary CNS lymphoma, T-cell lymphoma, EBV-positive lymphoma, corticosteroid-refractory, gross total resection, PCNS-TCL, EBER *in situ* hybridization

## Abstract

Primary central nervous system T-cell lymphoma (PCNS-TCL) is an exceptionally rare entity, representing less than 5% of all PCNSLs. Its diagnosis is frequently delayed due to nonspecific radiologic features and an often absent or poor response to corticosteroid therapy. Here, we present a unique case of an immunocompetent 58-year-old male with a solitary, EBV-positive, T-cell lymphoma localized in the right temporal lobe. The lesion was refractory to corticosteroid therapy and ultimately required gross total resection (GTR) due to progressive neurological deterioration. Postoperative histopathological examination confirmed a cytotoxic T-cell phenotype with EBV positivity. The patient demonstrated immediate neurological improvement post-surgery. This case underscores the importance of considering T-cell lymphoma in the differential diagnosis of solitary intracranial masses and suggests that surgical intervention may be warranted in select cases.

## Introduction

Primary CNS lymphoma is a rare but aggressive malignancy, with the vast majority being of B-cell origin. T-cell variants (PCNS-TCL) are exceedingly rare and are often associated with diagnostic delay and therapeutic challenges due to their non-specific clinical presentation and poor response to corticosteroids. In the largest dedicated series to date, Shenkier et al. reported 45 cases of PCNS-TCL across 12 institutions, highlighting the scarcity of this entity and its clinical similarities to B-cell PCNSL (1). Epstein–Barr virus (EBV) positivity, as seen in extranodal peripheral T-cell lymphoma not otherwise specified (NOS), has been increasingly recognized in these rare cases and is associated with particularly poor prognosis. This case is notable not only due to its rarity and EBV association but also because the patient’s deterioration despite high-dose corticosteroids necessitated a gross total surgical resection—an approach that deviates from traditional management strategies that prioritize biopsy. Our findings align with emerging literature suggesting a potential role for resection in select patients with solitary lesions, refractory symptoms, and accessible tumor locations (2, 3).

## Case description

A 58-year-old male presented to the emergency department with a one-month history of progressively worsening headaches. Neurological examination revealed no pathological findings.

His medical history was notable only for heavy tobacco use, with no known comorbidities or regular medication use. Initial head computed tomography (CT) with intravenous contrast revealed a right temporal intra-axial mass with relatively homogeneous contrast enhancement and extensive surrounding edema. High-dose oral dexamethasone was initiated to manage the edema while awaiting further diagnostic work-up.

**Figure 1:**
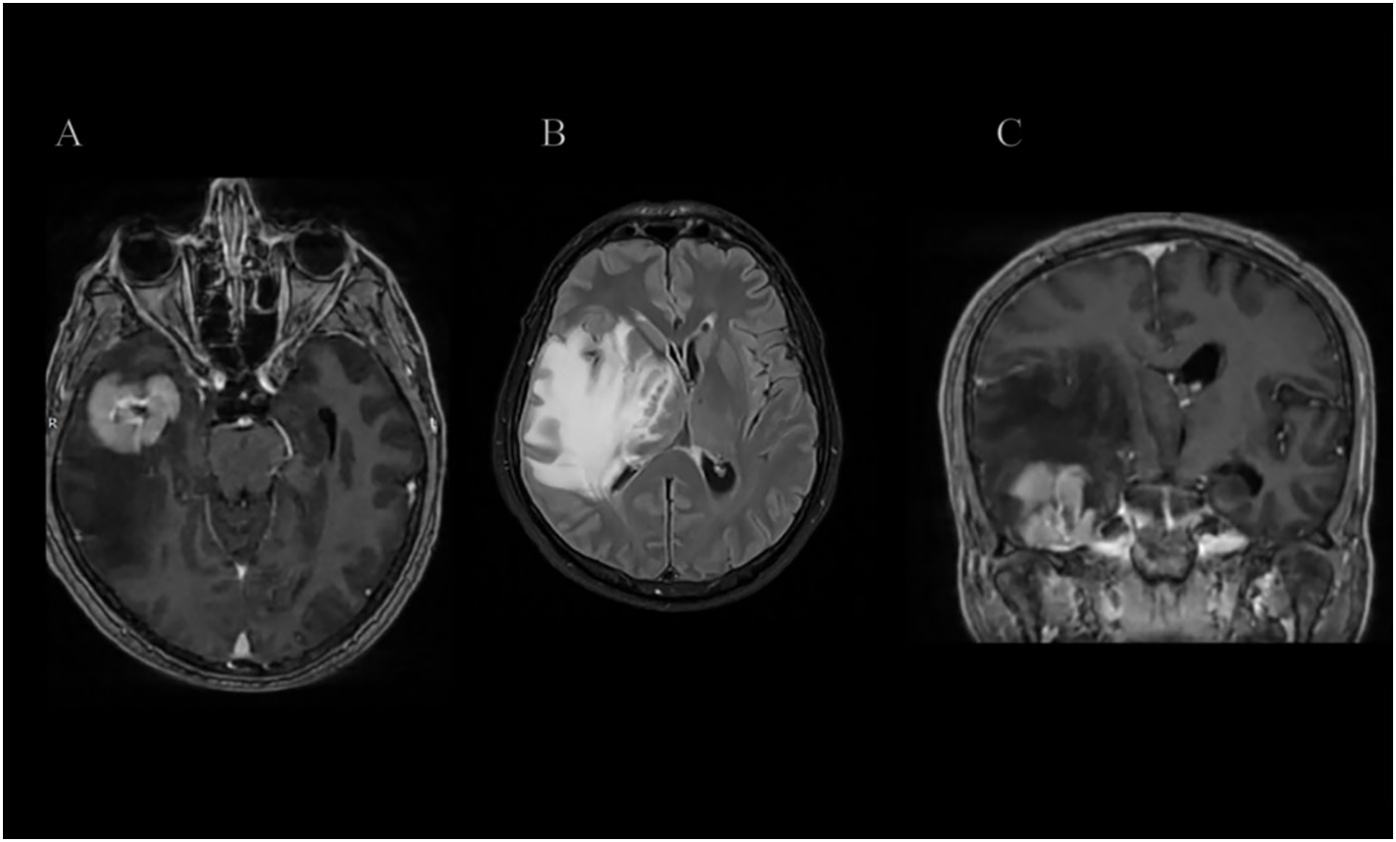
**(A–C)** Axial and Coronal T1-weighted post-contrast (T1 + IVC) and T2 FLAIR MRI sequences demonstrate a relatively homogeneously enhancing intra-axial mass centered in the right temporal lobe, predominantly involving the fusiform and inferior temporal gyri and extending into the underlying white matter. The lesion is associated with marked vasogenic edema, resulting in significant mass effect, midline shift, and effacement of the right crural cistern.

**Figure 2:**
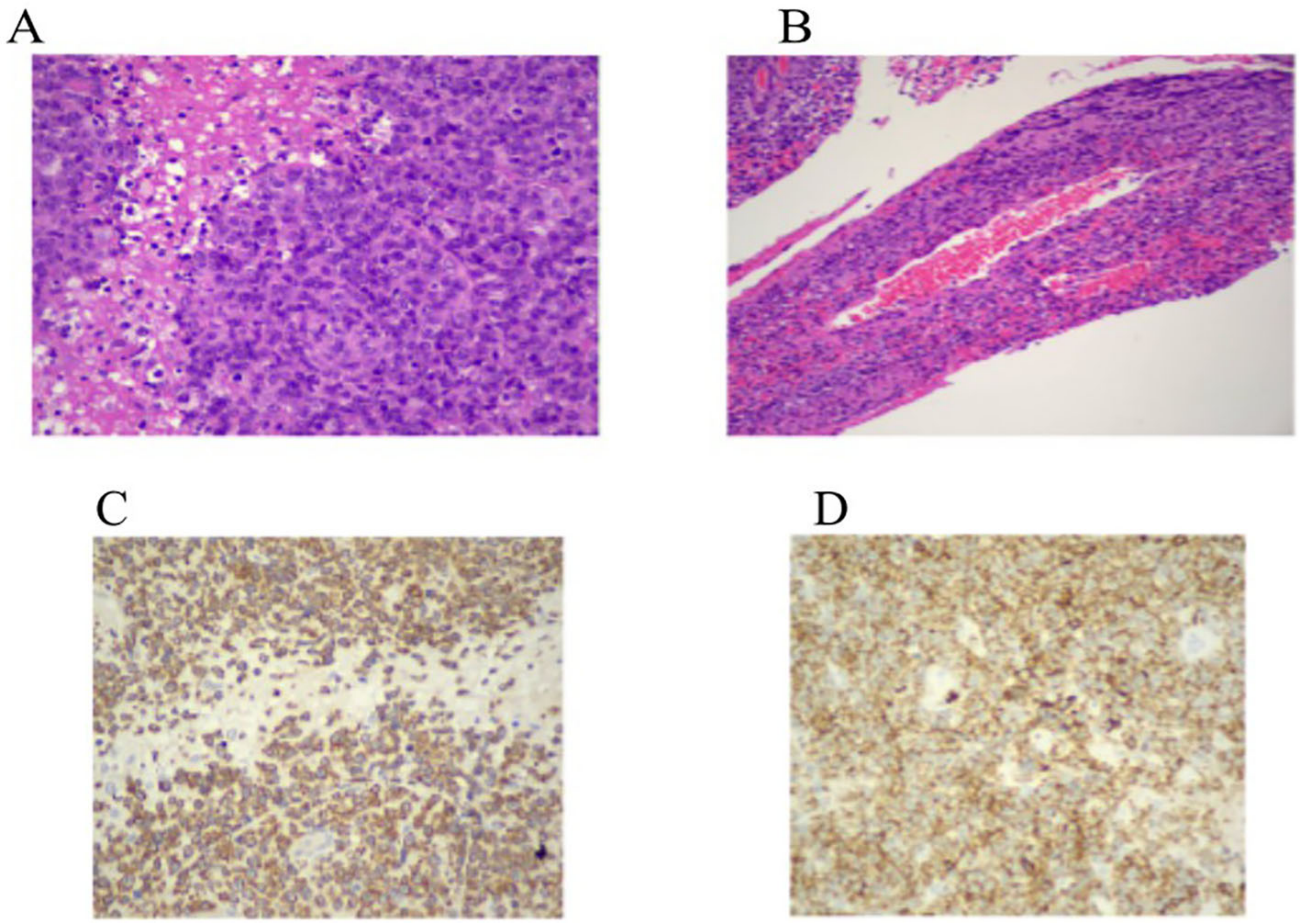
**(A, B)** Hematoxylin and eosin staining (×200, ×400) shows a dense in ltrate of large atypical lymphoid cells with prominent nucleoli, areas of necrosis, and a characteristic angiocentric pattern surrounding leptomeningeal vessels with associated brinoid vascular necrosis. **(C, D)** Immunohistochemistry for CD3 demonstrates strong membranous and cytoplasmic expression in the neoplastic cells. CD2 shows moderate membranous staining, supporting T-cell lineage.

**Figure 3:**
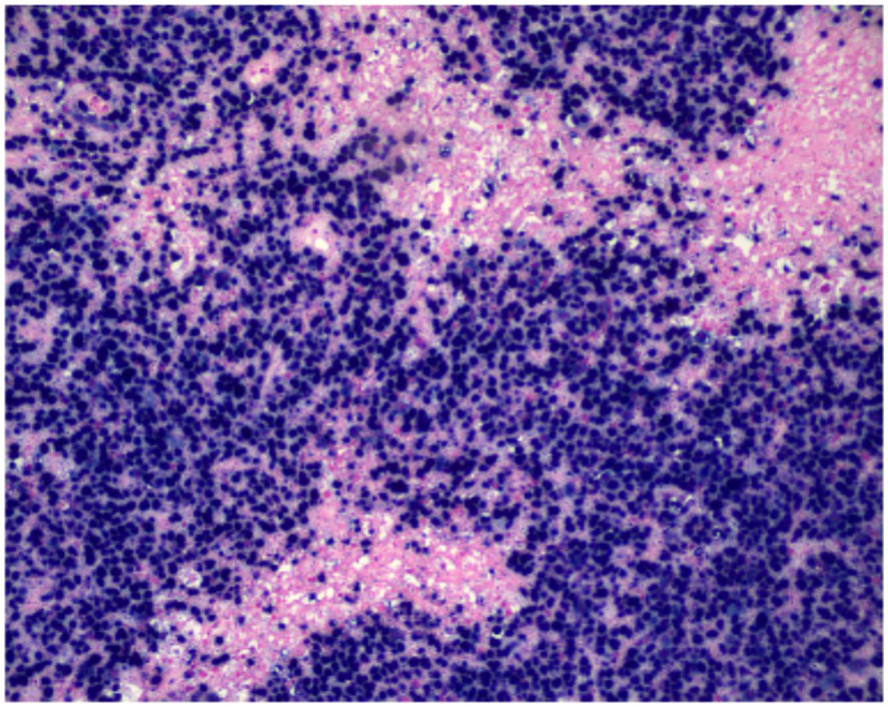
Nuclear positivity for EBV-encoded RNA is demonstrated by chromogenic in situ hybridization (CISH, ×200), indicating EBV involvement within the neoplastic lymphoid cell population.

Subsequent magnetic resonance imaging (MRI) confirmed the presence of a homogeneously enhancing mass in the right temporal lobe causing significant vasogenic edema as shown in **Figures 1**–**3**. Comprehensive systemic staging, including thoracic and abdominal imaging, and a negative PET Scan revealed no evidence of systemic or extracranial disease, raising suspicion for a primary central nervous system lesion.

### Clinical course and surgical intervention

Despite initiation of high-dose oral dexamethasone to reduce cerebral edema and mass effect during the preoperative period, the patient’s neurological status continued to decline. He was subsequently admitted to the neurosurgical ward and transitioned to intravenous corticosteroid therapy. Nevertheless, his condition progressively deteriorated, prompting the decision for semi-urgent surgical intervention.

### Surgical procedure

A right temporal craniotomy was fashioned to achieve broad exposure of the lesion and adjacent healthy brain parenchyma. Intraoperative frozen section analysis was suggestive of a lymphoproliferative process. Definitive histopathological evaluation, including immunohistochemistry, confirmed the diagnosis of primary central nervous system T-cell lymphoma (4, 5).

Given the patient’s poor clinical response to corticosteroids and existing literature suggesting a potential benefit of gross total resection (GTR) in selected cases, a complete supramarginal excision of the mass was undertaken (3, 6). The patient experienced marked postoperative neurological improvement and was subsequently referred to hematology/oncology for further management based on the definitive pathological diagnosis.

### Postoperative course

Three weeks postoperatively, the patient was admitted to the hematology-oncology unit and initiated on the AspaMetDex chemotherapy regimen (pegaspargase, methotrexate, and dexamethasone). During treatment, the patient developed acute kidney injury (AKI), which was managed conservatively with intravenous hydration, and hypofibrinogenemia, successfully corrected with fibrinogen supplementation. The regimen was otherwise well tolerated, and the patient was discharged in stable condition after a 2-week hospital stay.

Follow-up brain MRI at three months postoperatively showed no evidence of tumor progression or recurrence, either at the resection site or elsewhere in the central nervous system.

A visual timeline of the key clinical events from symptom onset through 3-month follow-up is presented in **Table 1**.

**Table 1:** Timeline of the clinical course from initial presentation to 3-month follow-up.

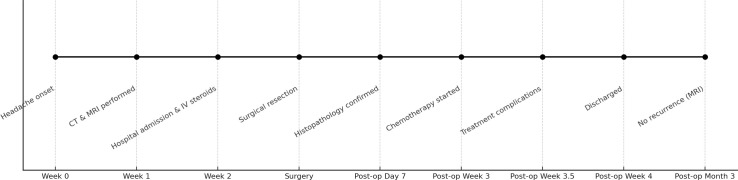	

Post-operative intervals are indicated on the x-axis.

### Histopathological and immunophenotypic findings

Microscopic examination of the resected specimen revealed a dense infiltrate of large atypical lymphoid cells with irregular nuclear contours, prominent nucleoli, and areas of necrosis. A striking feature was the angiocentric and angioinvasive pattern of infiltration, with tumor cells arranged concentrically around leptomeningeal blood vessels, accompanied by fibrinoid vascular necrosis.

Immunohistochemical analysis demonstrated strong membranous and cytoplasmic expression of CD3, confirming T-cell lineage. CD2 was moderately expressed, while CD4 was globally negative and CD8 was positive in only approximately 15% of the neoplastic cells. The tumor showed a cytotoxic immunophenotype, with strong expression of TIA-1 and Granzyme B, whereas Perforin was negative. CD56 and T-cell receptor gamma-delta (TCRγδ) were not expressed. The Ki-67 proliferation index was greater than 95%, indicating a highly proliferative tumor.

*In situ* hybridization for Epstein-Barr virus–encoded RNA (EBER) revealed strong and diffuse nuclear positivity in virtually all tumor cells, confirming EBV association. This case is not classified as a large T-cell lymphoma due to the uniform EBER positivity, cytotoxic immunophenotype, and characteristic angiocentric necrosis, findings consistent with an EBV-positive extranodal T-cell lymphoma.

Furthermore, this case does not meet criteria for a T follicular helper (TFH) phenotype lymphoma, as the neoplastic cells lack expression of defining TFH markers such as PD-1, ICOS, and BCL6, and do not exhibit a TFH-associated immunophenotype.

These morphological and immunophenotypic features support a diagnosis of extranodal EBV-positive peripheral T-cell lymphoma, presenting as a primary central nervous system (CNS) lymphoma, in accordance with the 2022 WHO classification (ICD-O 9702/3).

EBV serology demonstrated positive viral capsid antigen (VCA) IgG and Epstein–Barr nuclear antigen (EBNA) IgG, with negative VCA IgM, consistent with prior EBV infection. This serological profile, which reflects past exposure, is the most common pattern observed in immunocompetent adults and is typical even in cases of EBV-associated lymphomas.

## Discussion

Primary central nervous system lymphoma (PCNSL) is a rare neoplasm, comprising approximately 2–3% of all brain tumors, with the vast majority representing diffuse large B-cell lymphoma (DLBCL) variants. In contrast, T-cell PCNSL (PCNS-TCL) is exceptionally rare, accounting for less than 5% of all PCNSLs, and is often underrecognized due to its atypical presentation and nonspecific imaging features (4, 5).

In the presented case, the patient developed a solitary, homogeneously enhancing temporal mass with associated vasogenic edema—radiological features commonly seen in high-grade gliomas or metastatic disease. The absence of systemic disease on staging investigations raised suspicion for a primary CNS process. The diagnosis was ultimately established through intraoperative frozen section analysis suggestive of lymphoma, and later confirmed via immunohistochemistry as a T-cell lymphoma. This sequence reflects the central role of histopathological evaluation in differentiating PCNSL subtypes, as imaging alone cannot reliably distinguish between B- and T-cell origins (3). Menon et al. reported the largest Western series of PCNS-TCLs (n=18), highlighting the predominance of cytotoxic immunophenotypes and the absence of EBV in all cases, which contrasts with our EBV-positive tumor (7).

One of the unique aspects of this case is the lack of response to high-dose corticosteroids, which are typically effective in reducing tumor burden and peritumoral edema in B-cell PCNSL. Literature suggests that T-cell variants may demonstrate reduced corticosteroid sensitivity, potentially due to variations in glucocorticoid receptor expression or inherent resistance pathways such as Bcl-2 overexpression (8–11). The patient’s clinical deterioration despite both oral and intravenous corticosteroids necessitated semi-urgent surgical intervention.

The role of surgical resection in PCNSL has historically been limited to diagnostic biopsy due to the diffuse nature of the disease and its known radiosensitivity and chemosensitivity. However, emerging evidence—particularly from series involving B-cell PCNSL—suggests that gross total resection (GTR) may confer survival benefit in selected cases with solitary, accessible lesions and deteriorating neurological status (2, 9). Data from the German PCNSL Study Group-1 trial indicated that patients undergoing subtotal or gross total resection had improved progression-free and overall survival compared to those who had only biopsies (2).Additionally, a systematic review highlighted that, in selected patients with solitary, accessible lesions, GTR could be performed safely and might offer survival benefits. While data specific to PCNS-TCL are limited, the poor corticosteroid response and solitary nature of the lesion in this case justified a more aggressive surgical approach. Notably, the patient improved significantly following supramarginal GTR, further supporting this individualized management strategy.

### Comparative case analysis

A review of the published literature reveals several case reports and small series that collectively underscore the heterogeneity and clinical challenges of managing PCNS-TCL. **Table 2** summarizes key characteristics from selected cases, including patient demographics, lesion location, immunocompetence status, interventions, histology, corticosteroid response, imaging features, and outcomes.

**Table 2:** Summary of selected reported cases of primary CNS T-cell lymphoma, including patient demographics, lesion location, immunocompetence status, interventions, histology, corticosteroid response, imaging features, and outcomes.

Reference	Patient Age/Sex	Location	Immunocompetent	Intervention	Histology	Outcome	Corticosteroid response	Imaging features
Bednar et al. (4)	36/M	Cerebellum	Yes	Subtotal resection + radiotherapy	T-cell lymphoma	Alive at 36 months	Not specified	Homogeneous enhancement
Takeshita et al., (20)	46/M	Right parietal lobe	Yes	Biopsy + chemoradiotherapy	T-cell lymphoma	Well at 24 months	Not specified	Homogeneous enhancement
Kawamura et al., (21)	16/M	Cerebral hemisphere	Yes	Gross total resection + chemotherapy	Ki-1 (CD30+) T-cell lymphoma	Deceased at 6 months	Poor response	Enhancing mass
Bird et al. (3)	61/F	Left frontal lobe	Yes (Sjögren’s)	Open biopsy	Peripheral T-cell lymphoma	Outcome not specified	Not specified	Contrast-enhancing lesion
Ferreri et al. (16)	Multiple	Various	Mostly Yes	Various (mostly biopsy)	Peripheral T-cell lymphoma	Median DSS: 25 months	Variable	Various
Goldbrunner et al. (13)	16/M	Cerebral hemisphere	Yes	Gross total resection + chemotherapy	Ki-1 (CD30+) T-cell lymphoma	Deceased at 6 months	Poor response	Enhancing hemispheric mass
Rigoni et al. (8)	61/F	Not specified	Yes	Biopsy	T-cell lymphoma	Outcome not specified	Not specified	Mimicking autoimmune encephalitis
Alhosaini et al. (5)	43/M	Not specified	No (AIDS)	Stereotactic biopsy + chemoradiotherapy	T-cell lymphoma	Outcome not specified	Not specified	Not specified
Guzzetta et al. (9)	82/M	Spinal cord	Yes	Open biopsy	T-cell lymphoma	Outcome not specified	Not specified	Not specified
Kang et al. (6)	Not specified	Not specified	Not specified	Biopsy	NK/T-cell lymphoma	Outcome not specified	Not specified	Leukoencephalopathy-like pattern

Notably, gross total resection (GTR) was performed in a minority of cases—primarily in younger patients with solitary, accessible lesions—such as in the reports by Kawamura et al. and Goldbrunner et al. (12, 13), both involving adolescent males. Despite surgical intervention, these patients had poor survival, highlighting the aggressive biology of certain T-cell subtypes like Ki-1 (CD30+) lymphoma. On the other hand, Takeshita et al. reported a favorable 24-month outcome in a middle-aged male treated with biopsy and chemoradiotherapy alone, suggesting that multimodal therapy remains the cornerstone of treatment when feasible.

The table also emphasizes the variability in imaging findings and corticosteroid responsiveness, with several entries lacking detailed documentation. However, poor steroid responsiveness—similar to our case—is recurrently noted in cases with aggressive phenotypes and EBV association. Notably, Kang et al. (6) described a case of primary CNS NK/T-cell lymphoma, a subtype that is by definition associated with EBV positivity according to the 2022 WHO classification. Although EBER status was not explicitly stated in their report, the diagnosis of NK/T-cell lymphoma implies clonal EBV infection, as EBV is a defining feature of this entity. This makes their case, alongside ours, one of the very few reported instances of EBV-positive PCNS-TCL in the literature.

Together, these cases, although limited by heterogeneity and incomplete data, suggest that individualized management based on lesion accessibility, histologic subtype, and EBV status is critical. Our case adds to this evolving body of literature by demonstrating a rare EBV-positive T-cell phenotype with favorable early postoperative recovery following GTR, reinforcing the potential role of surgery in selected scenarios.

Prognostically, PCNS-TCL appears to have a less favorable outcome compared to its B-cell counterpart, with reported median overall survival ranging from 12 to 25 months, depending on treatment and patient factors (1, 14–16). Factors influencing prognosis in PCNS-TCL include patient age, performance status, extent of CNS involvement, treatment responsiveness, and EBV status. As shown in the largest series to date by Shenkier et al., performance status and the use of methotrexate-based regimens are independently associated with improved survival, reinforcing their central role in management despite the lack of EBV-specific treatment protocols.

More specifically Epstein–Barr virus (EBV) positivity in peripheral T-cell lymphomas, with CNS involvement, is increasingly recognized as a marker of aggressive disease biology and poor prognosis. According to the 5th edition of the WHO classification (2022), EBV-positive peripheral T-cell lymphoma (ICD-O 9702/3) constitutes a distinct clinicopathologic entity, often associated with rapid progression, extranodal involvement, and resistance to standard chemotherapy regimens such as CHOP. Alaggio et al. provide the updated WHO framework that formally distinguishes EBV-positive peripheral T-cell lymphomas, including γδ variants, emphasizing their aggressive behavior and extranodal predilection (17). In particular, CNS involvement further compounds the adverse outlook, with reported median overall survival frequently less than 12 months despite aggressive therapy (12, 18). The presence of EBV within tumor cells, confirmed by EBER *in situ* hybridization, may prompt consideration of alternative treatment strategies, including high-dose methotrexate-based regimens for CNS penetration, L-asparaginase–containing protocols (e.g., SMILE), or even early consolidation with autologous stem cell transplantation. In select cases, investigational therapies targeting immune checkpoint pathways (e.g., PD-1 inhibitors) have been explored, given the frequent expression of PD-L1 in EBV-positive lymphomas (19). These findings highlight the importance of establishing EBV status not only for diagnostic classification but also for prognostic stratification and potential modification of management.

### Strengths and limitations

Strengths:

This case contributes to the scarce literature on EBV-positive primary CNS T-cell lymphoma (PCNS-TCL) in immunocompetent patients, offering a detailed diagnostic and therapeutic account aligned with current WHO classification. It highlights the potential role of gross total resection (GTR) in corticosteroid-refractory cases with solitary, accessible lesions, supporting the value of individualized, multidisciplinary management.

Limitations:

The rarity of EBV-positive PCNS-TCL limits generalizability, and current management strategies are based on heterogeneous, low-level evidence. While GTR led to clinical improvement here, its broader role remains unclear due to the absence of prospective data. Similarly, the optimal systemic treatment for EBV-driven CNS T-cell lymphomas remains undefined, underscoring the need for more robust clinical evidence.

## Patient perspective

The patient noted significant improvement in neurological symptoms following surgery and expressed appreciation for the rapid intervention. He was actively involved in discussions regarding his diagnosis and treatment plan and demonstrated a clear understanding of the therapeutic strategy and its rationale.

## Conclusion

This case underscores the importance of considering T-cell lymphoma in the differential diagnosis of solitary CNS lesions, especially in cases unresponsive to corticosteroids. While biopsy remains the standard diagnostic approach, gross total resection may offer therapeutic benefit in selected patients with progressive symptoms and localized disease. Early recognition, prompt histopathological diagnosis, and timely initiation of aggressive multimodal therapy are essential to improving outcomes in patients with EBV-positive peripheral T-cell lymphoma involving the central nervous system. Given the aggressive nature and poor prognosis associated with this entity, particularly when EBV-driven, even a short delay in treatment can significantly impact survival. As such, clinicians should maintain a high index of suspicion when evaluating rapidly progressive CNS lesions, especially in immunocompromised or high-risk individuals. Definitive diagnosis requires tissue biopsy with EBV-specific testing, such as EBER *in situ* hybridization, to guide classification and tailor therapeutic strategies. Early referral to specialized hematology-oncology services for systemic chemotherapy, CNS-directed therapy, and consideration of stem cell transplantation is imperative to optimize clinical outcomes in this rare and highly aggressive malignancy. Given the rarity of PCNS-TCL, further studies are needed to better define its optimal management.

## Data Availability

The original contributions presented in the study are included in the article/supplementary material. Further inquiries can be directed to the corresponding author.
